# Effects of Docosahexaenoic Acid on Prostate Cancer

**DOI:** 10.3390/jox15040111

**Published:** 2025-07-04

**Authors:** Guilherme Henrique Tamarindo, Gustavo Matheus Amaro, Alana Della Torre da Silva, Rejane Maira Góes

**Affiliations:** 1 Institute of Biology, State University of Campinas (UNICAMP), Campinas 13083-862, São Paulo, Brazil; guilherme.tamarindo@lnbio.cnpem.br; 2 Brazilian Biosciences National Laboratory (LNBio), Brazilian Center for Research in Energy and Materials (CNPEM), Campinas 13083-100, São Paulo, Brazil; 3 Department of Biological Sciences, Institute of Biosciences, Humanities and Exact Science, São Paulo State University (UNESP), São José do Rio Preto 15054-000, São Paulo, Brazil; gustavo.amaro@unesp.br (G.M.A.); della.torre@unesp.br (A.D.T.d.S.); 4 Department of Clinical Analysis, School of Pharmaceutical Sciences in Araraquara, São Paulo State University (UNESP), Araraquara 14800-700, São Paulo, Brazil

**Keywords:** prostate cancer, PUFAs, lipids, DHA, omega-3, fish oil

## Abstract

The polyunsaturated fatty acids of the omega-3 class have been widely investigated due to their antitumor properties, including in prostate cancer (PCa). Among them is docosahexaenoic acid (DHA, C22:6 ω-3), whose biological activity is higher than other omega-3s, exhibiting a stronger impact on PCa. The specific mechanisms triggered by DHA are blurred by studies that used a blend of omega-3s, delaying the understanding of its biological role, and hence alternative therapeutic approaches. DHA is differentially processed between normal and malignant epithelial PCa cells, which suggests its function as a tumor suppressor. At cell-specific level, it downregulates key pathways in PCa, such as androgen signaling and lipid metabolism, but also changes membrane composition by disrupting phospholipid balance and increasing unsaturation status, arrests the cell cycle, and induces apoptosis and reactive oxygen species (ROS) overproduction. At the tissue level, DHA seems to influence stromal components, such as the inhibition of cancer-associated fibroblast differentiation and resolution of inflammation, which generates a microenvironment favorable to PCa initiation and progression. Considering that such effects are misunderstood and assigned to omega-3s in general, this review aims to discuss the specific effects of DHA on PCa based on in vitro and in vivo evidence.

## 1. Introduction

**Figure 1:**
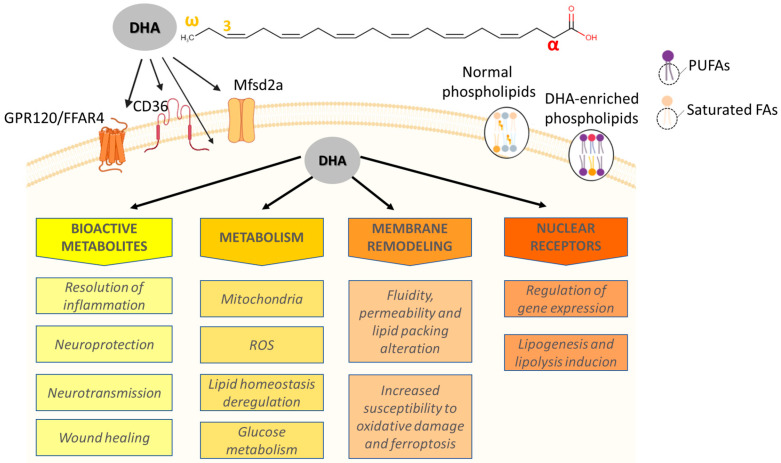
Summary of DHA mechanisms. DHA may enter the cells by diffusion or through membrane receptors GPR120/FFAR4, CD36, and Mfsd2a. Once inside the cells, the omega-3 is likely to trigger downstream biological events, affecting many pathways whose outcome depends on the cell type, its molecular context, and DHA concentration. Legend: DHA—docosahexaenoic acid; ROS—Reactive oxygen species; FA—fatty acids; PUFAs—polyunsaturated fatty acids; ω—omega extremity; α—alpha carbon; GPR120—G-protein coupled receptor 120; FFAR4—Free Fatty Acid Receptor 4; Mfsd2a—Major Facilitator Superfamily Domain containing 2a.

In the last decade, the polyunsaturated fatty acids (PUFAs) belonging to the omega-3 class have been in the spotlight due to their properties in several healthy conditions, such as brain function improvement [1], serum lipid profile normalization [2], liver injury mitigation [3], and a decrease of cardiac pathologies [4], but also in pathological conditions, such as growth suppression of distinct tumors [5,6,7,8,9]. These highly unsaturated long-chain fatty acids have the first double bond located on the third carbon from the omega extremity, being the alpha carbon the first after the carboxyl group, as shown in Figure 1 [10]. The most abundant omega-3 is alpha-linolenic acid (ALA, C18:3 ω-3), found in edible plants, particularly in some seeds, being the precursor for longer and more unsaturated fatty acids such as eicosapentaenoic acid (EPA, C20:5 ω-3) and the docosahexaenoic acid (DHA, C22:6 ω-3) [11]. This conversion is lower in men than women [10,12], with diet becoming the main source through the intake of marine cold fishes, eggs, algae, or nutritional supplementation [10]. Such a condition entails that omega-3s are mostly obtained as a blend and not as isolated compounds, reflecting many experimental and epidemiological studies that investigated DHA and EPA together, even though the ratio was emphasized [13,14,15]. Despite the beneficial effects of these fatty acids, increasing evidence has shown that each of the omega-3s seems to play a different role in health and disease, making it difficult to determine their individual contribution to a given outcome.

Compared to EPA, DHA has many distinct effects on health, such as on heart function, protein synthesis, inflammation, lipoprotein metabolism, hemodynamics, vascular function, and insulin signaling [16]. In the human body, DHA is found mostly in cell membranes and is crucial to the function of many organs, including the heart, eyes, skeletal muscles, testis, and liver [10,11,17]. Especially in the brain, DHA is required for optimum development, comprising 10–20% of the brain’s total fatty acids and 90% of its omega-3 content, which is closely related to synapse functioning and the transmission of electric pulses [18]. Thus, DHA supplementation may improve long-term neurodevelopmental outcomes in offspring, particularly in women with pregnancy complications and altered placental transport [19]. Concerning the male reproductive system, there is a specific DHA requirement in the testis for normal spermatogenesis, and it is essential for sperm motility [20] due to acrosome biogenesis via modulation of protein traffic from the Golgi complex, necessary for proacrosomal granule fusion [21]. However, DHA functions in other tissues, such as the prostate gland under healthy and pathological conditions, are not fully elucidated. In prostate cancer (PCa), experimental evidence described its antiproliferative and apoptotic action on tumor cells in distinct stages of the disease progression [22,23,24,25,26]. This is of particular interest since PCa is among the five leading causes of death in men worldwide [27,28], and the available therapies may lead to recurrence and a more aggressive stage, such as the castration-resistant phenotype [29], a scenario that demands alternative approaches. Therefore, considering the growing evidence of DHA’s antitumor effects and its unclear role, we aimed to review its specific implications for PCa, retrieved from epidemiologic and experimental studies performed in both cell culture and animal models. In the present review, we discuss the possible role of DHA as a tumor suppressor and put together the mechanisms whereby DHA may exert its effects. We also highlight the controversies in clinical findings that correlate or not this omega-3 with the risk of PCa. Additionally, we identify biased results and gaps in the literature that can turn into opportunities for further investigation as well as current translational approaches that could benefit from DHA administration.

## 2. Overview of Molecular Aspects, Metabolism, and Sources

DHA, rarely referred to as cervonic acid, is formally known as *cis*-4,7,10,13,16,19-docosahexaenoic acid and has a 22-carbon chain with 6 *cis* double bonds starting at the third carbon of the omega extremity (Figure 1) [10]. Despite having been considered an essential fatty acid in the past, DHA may be synthesized from ALA at low rates in humans through elongations and desaturations that occur in the endoplasmic reticulum and peroxisomes [10,12,18]. This is supported by evidence that increased ALA intake does not exert a strong effect on DHA serum levels, corroborating that its main source is from diet [18,30]. Indeed, specific diets increase DHA levels in serum, such as those based on seafood [31]. Despite most of the omega-3 coming from diet, it is important to mention that its blood levels are also determined by genetic variations of the fatty acid desaturase (*FADS*) and fatty acid elongase (*ELOVL*) genes [32]. This is due to their role in producing long-chain fatty acids from PUFAs with shorter carbon chains taken up from the diet [32]. This ability can vary due to single nucleotide polymorphisms (SNPs) in FADS1 (rs174533), FADS2 (rs174575 and rs498793), and *ELOVL2* (rs2236212) [32], which affects only DHA but not EPA levels. Once ingested, DHA is processed by the gastrointestinal tract and carried out to the bloodstream as a component of lipoproteins, being accumulated into adipocytes or used by other tissues, as already mentioned [10]. DHA can enter the cells mainly through GPR120 (G-Protein Coupled Receptor 120) and Mfsd2a (Major Facilitator Superfamily Domain Containing 2a), a G-coupled protein and a sodium-dependent transporter, respectively (Figure 1), but other receptors have also been investigated, such as CD36 [33,34,35,36,37,38,39,40,41]. Especially in GPR120, it triggers several types of downstream signaling, including stimulation of cytosolic phospholipase A2 (cPLA2) and prostaglandin-endoperoxide synthase 2 (PTGS2), an increase in intracellular calcium concentration, and inhibition of TLR2/3/4 (Toll-Like Receptor 2, 3 and 4) and a TNF-α (Tumor Necrosis Factor alpha) pro-inflammatory cascade in addition to TAK1 (Transforming growth factor beta-activated kinase 1) through β-arrestin2/TAB1 (TAK1-binding protein 1) [35,36,42,43]. DHA is also a known ligand of the peroxisome proliferator-activated receptors (PPARs), which are transcription factors that regulate cell proliferation, apoptosis, adipogenesis, lipid metabolism enzymes, mitochondrial biogenesis and function, and other metabolism-related genes [44,45,46,47]. Moreover, DHA is incorporated into glycerophospholipids, such as phosphatidylethanolamine, phosphatidylserine, and phosphatidylcholine, in addition to altering membrane systems, including mitochondria and the endoplasmic reticulum [25,48,49].

Although this review does not aim to discuss the PUFAs and DHA metabolism itself since this has already been done [50,51], it is important to mention that bioactive lipids derived specifically from DHA are formed via enzymatic pathways that oxidize it into mono-, di-, and tri-hydroxy-DHA, and epoxy- and oxo-DHA, or modify it by a free radical non-enzymatic mechanism [50]. DHA catabolism is exerted by PTGS2 as well as 5-, 12-, and 15-lipoxygenase (LOX), forming hydroperoxides that serve as substrates for hydrolases, glutathione S-transferase, and members of the cytochrome P450 superfamily (epoxydases, ω-hydrolases) [10,52,53,54]. Also, degradation of DHA metabolites may be mediated by oxidation, conjugation with glutathione (GSH), or hydrolysis by fatty acid amide hydrolase as well as epoxide hydrolase [54,55,56]. DHA metabolism involves the formation of several potent signaling lipids, such as maresins, protectins, epoxides, neuroprostanes related to biological outcomes like antiproliferation, inflammatory modulation, lipoperoxidation, cardiac function, and antitumor [50]. DHA and its metabolites have many mechanisms described in the literature and are summarized in Figure 1, varying according to cell type and between normal and pathological conditions. This indicates that this is a complex fatty acid exhibiting a plethora of actions which have received recent attention, and here we will emphasize those related to PCa.

## 3. DHA Levels in Normal and PCa Cells: Evidence for a Tumor Suppressor?

Differences in the levels of naturally occurring DHA have been described in the literature between normal and prostate cells. Without DHA supplementation in the medium, RWPE-1 benign cells naturally exhibit intracellular levels higher than those found in malignant PC3: 2.58 ± 0.49 and 0.71 ± 0.03%, respectively [26]. Once supplemented in the medium, DHA levels increase in both non-tumor and PCa cells’ fatty acid milieu [26] and can change the *sn*-2 position of the glycerol backbone, thereby changing the species of phospholipids [57]. PUFAs are known to induce membrane lipoperoxidation due to their high unsaturation levels, which leads to two possible scenarios. First, it was hypothesized that benign cells have low tolerance for PUFAs, DHA included, because they already have their own pool in addition to showing a glycolytic phenotype over fatty acid oxidation. Normal prostate cells have decreased mitochondrial activity regulated by androgens to sustain the secretory function of citrate, forcing them into the glycolytic pathway to supply bioenergetic demands. Since DHA is incorporated into phospholipids, its supplementation may disrupt lipid membrane status by increasing unsaturation, hence susceptibility to oxidative stress. Despite this still requiring investigation, it would be in line with studies showing the stronger effect of DHA on PNT1A growth, a non-tumor cell line [9,24,25]. Secondly, there is evidence that PCa cells have increased saturated fatty acid synthesis [58,59,60], and their uptake drives PCa [61], while PUFAs induce ferroptosis [62]. Additionally, PCa cells already have increased oxidative stress [63,64]. Therefore, it is reasonable to consider that PCa cells avoid PUFAs, which may sensitize them to oxidative damage. Indeed, DHA has been shown to increase sensitivity to oxidative stress involving the Nuclear Factor Kappa beta (NFkβ) [65]. The protective role of DHA is also supported by findings that unmethylation of NUDT21 (Nudix Hydrolase 21) decreases its level and contributes to resistance to enzalutamide in PCa cells [66].

Despite this body of evidence being correlative, it seems that DHA acts as a tumor suppressor. However, there is a lack of validation in the literature to support this hypothesis, and further studies are required to better elucidate this issue. As we show next, this association remains elusive when combining epidemiologic with experimental data.

## 4. Epidemiologic Findings on DHA Association with PCa Risk

Despite increasing evidence concerning DHA’s antiproliferative properties, some studies have suggested an increase in serum levels of DHA with a high risk of PCa, while others have reported this correlation as negative or at least inconsistent [67,68,69,70,71,72,73,74,75]. By using liquid chromatography/mass selective detector separation and logistic regression analysis, it was reported that DHA levels were elevated in lipid extract from red blood cells after fasting [67]. The study evaluated men undergoing radical prostatectomy over 35 years old, with a Gleason sum ≥ 6, clinical ≥ T1c, and PSA ≥ 4ng/mL, and free of 5-alpha reductase inhibitors treatment, suggesting an association with high-grade PCa [67]. Such an association was also described in men diagnosed with incident primary PCa from the SELECT Trial [68] as well as in patients from the Prostate Cancer Prevention Trial (non-fasting blood collection), which evaluated (using gas chromatography) inflammation-related phospholipid fatty acids in a nested case–control analysis of participants with 55–84 years old [69]. This study showed a positive, but not linear, association between DHA levels in the serum (proportion to total fatty acids) and the risk of high-grade PCa [69]. One limitation acknowledged by the authors is that they used only localized PCa and high-gradestage, defined as a Gleason score of 8–10 [69]. In a meta-analysis of five studies, there was reported a curvilinear association of PCa risk with DHA intake (g/day), but the authors did not estimate a linear trend [76]. This association was not found for EPA [76]. When assessing blood levels (nine other studies were evaluated), it seems that high concentrations of both DHA and EPA can increase PCa risk [76]. In a study limited to the Jamaican male population, DHA levels alone (assessed using gas chromatography) were positively correlated to PSA, but when combined with other fatty acids, they may be associated with an increase or decrease of tumor volume, as in the case of linoleic acid and arachidonic acid, respectively [71]. However, the association between serum DHA or total omega-3 (after 12 h fasting) and PSA was either nonexistent or quite weak in another clinical study using gas chromatography to assess samples restricted to non-Hispanic white men over 40 years old [72]. Sorongon-Legaspi et al., in a meta-analysis restricted to the western population, found that both DHA and EPA blood levels had a positive association with high-grade prostate tumor risk, but only after adjustment of interstudy variability [73]. Although epidemiologic and systemic analyses have indicated a positive association between increased DHA levels and PCa risk, this is grossly inconsistent in the literature and needs careful interpretation. This is based on additional studies that did not find a positive association between DHA and PCa, but did find concerning other fatty acids [77,78,79]. Norrish et al. [75] reported high DHA levels in erythrocyte phosphatidylcholine associated with reduced PCa risk in New Zealand using age-matched community controls (men with 40–80 years old) and gas–liquid chromatography. Leitzman et al. also found that EPA and DHA intake were related to lower PCa risk [74], but a relevant limitation is that the authors assessed only the patient questionnaire. Evidence from a study with Jamaican men, which showed one of the highest PCa incidence rates in the world, also revealed an inverse correlation between DHA and PSA when combined with other PUFAs [71]. Additionally, the lack of concordance regarding DHA association with PCa may be due to limited populational studies that do not mirror heterogeneity, small number of assessed samples, technical limitations of fatty acid detection, biased interpretations due to patient background (such as lifestyle or patient diet), combined intake of DHA with other omega-3/omega-6 PUFAs, questionnaire-based studies only without appropriate controlled intake, absence of multicentric cohorts, biased reports in meta-analysis, limited association with PCa stage or Gleason score, and other factors. Also, it is worth mentioning that these epidemiologic data relied on the simple effect of omega-3 intake but did not consider the genetic variation (such as desaturases and elongases SNPs mentioned) in the subjects’ population which can sharply impact PUFA metabolism, hence the risk of PCa. Most of the studies available were published more than ten years ago, which calls for new investigations using more accurate methods, controlled studies, and assessment of changes in men’s quality of life. Finally, while epidemiological data suggest a positive association between high levels of serum DHA and PCa, most in vitro and in vivo studies support the antiproliferative role of this omega-3 or even its ability to serve as a co-adjuvant in chemotherapy, as further discussed. Therefore, DHA’s role, defined from epidemiologic evidence, in prostate carcinogenesis remains inconclusive and is still an open question that requires refinement and appropriate analysis.

## 5. Experimental Evidence on DHA’s Effect Against PCa

### 5.1. Androgen Signaling

**Figure 2:**
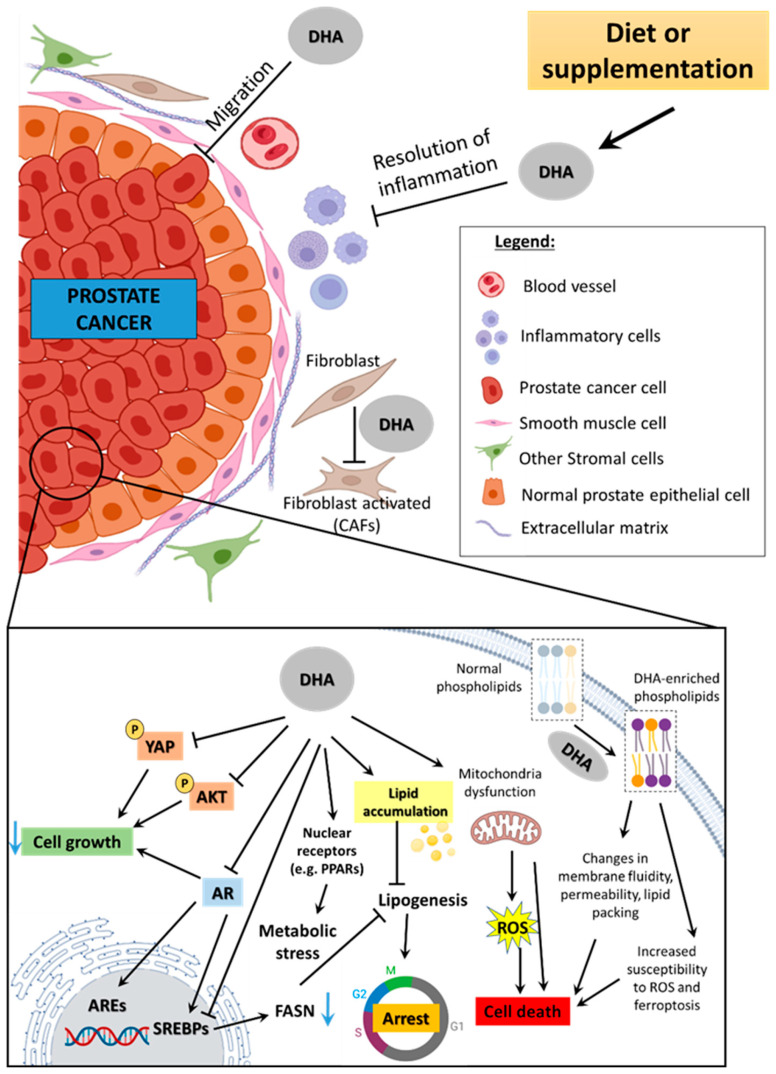
DHA’s specific effects on prostate cancer. DHA obtained through diet or supplementation reaches prostate cancer and may act at the tissue level by decreasing cell migration, resolving inflammation, or inhibiting fibroblast activation. At the cell level, the omega-3 suppresses cell growth via different pathways depending on the androgenic background. In AR-positive cells, it leads to AR degradation through the proteasome, which downregulates AREs and SREBPs, affecting lipid metabolism by decreasing FASN, hence lipogenesis. Also, DHA dysregulates mitochondria and induces ROS overproduction, which is closely related to cell death. Increase in DHA uptake is harmful to the cells because the rise in unsaturation status in their membrane sensitizes them to oxidative damage, but also changes membrane properties related to cell signaling, lipid packing, fluidity, and permeability. Legend: DHA—docosahexaenoic acid; AR—androgen receptor; ROS—reactive oxygen species; AREs—androgen-responsive elements; SREBPs—sterol regulatory element-binding proteins; FASN—fatty acid synthase; CAFs—cancer-associated fibroblasts; YAP—transcriptional coactivator Yes-associated protein; P—phosphate.

The prostate is an accessory gland of the male reproductive tract regulated by steroid hormones, mainly androgens, through specific receptors that, under normal conditions, control proliferation, cell death, metabolism, and secretory function [80,81]. This regulation by androgens is a pitfall that has driven therapeutic strategies in PCa [82,83]. In this line, DHA has been reported to affect androgen receptor (AR) signaling via different mechanisms. Mitsuhashi et al. [84] showed that unsaturated fatty acids, including DHA, decreased binding to the synthetic androgen R1881. Such regulation was further supported by evidence that such omega-3 repressed androgen-regulated gene expression, such as *KLK3*, *ODC*, *TMPRSS2*, *NKX3-1*, and *FKBP51* [49,85,86]. Hu et al. showed that DHA decreased cell proliferation associated with proteasome-mediated AR degradation in androgen-responsive LNCaP cells, whereas *AR* gene transcription was unchanged [85]. This is in line with a previous report that DHA prevented PCa progression to the androgen-independent stage due to AR expression decrease associated with AKT/mTOR modulation [87]. AR regulation by DHA was also described for RWPE-1 benign cells that showed decreased proliferation linked to the receptor reduction in addition to estrogen receptor alpha (ERα) [88]. However, DHA effects at early stages of PCa are controversial since unchanged AR expression was reported in the PNT1A cell line [24], which is non-tumor but has several malignant alterations [89]. As shown in Table 1, most of the genes modulated by DHA in prostate cell lineages are responsive to androgens. Thus, by regulating the expression of AR at the gene and protein levels, DHA indirectly modulates the expression of genes involved in different cancer hallmarks (Table 1). However, it is important to mention that other mechanisms towards proliferation inhibition might be triggered by DHA since it also decreased the growth of androgen-independent cells that do not express AR, such as PC3 [9]. This suggests that AR signaling is affected by DHA, but it has distinct targets according to PCa androgenic background, as shown in Table 1 and Figure 2.

DHA effects on androgen signaling were elucidated based mostly on in vitro studies. To date, animal studies using DHA free of additional fatty acids are scarce, mostly because its main source is fish oil-derived supplements composed of a combination of lipids. Therefore, animal investigations did not isolate the influence of DHA but considered its combination with EPA at different ratios, a bias that is also seen in epidemiologic and clinical trials, as already mentioned. This experimental approach is tricky because, on the one hand, it is closer to the human nutritional scenario, since those fatty acids are not ingested alone, but, on the other, it does not allow elucidation of the isolated role of each omega-3. Omega-3 consumption (5%) reduced estradiol, testosterone, and AR levels, promoting apoptosis and suppressing cell proliferation in C3(1) Tag mice [90]. Also, administration of seal oil (mixtures of 12% DHA, 18% EPA) to rats decreased benign prostatic hyperplasia (BPH) and AR expression in tissue, which was related to the DHA effect, also observed in assays with RWPE-1 cells [88]. The AR degradation mediated by proteasome activity was also observed in castration-resistant PTEN-null PCa mouse models [91]. Results from our research group also showed that a DHA-enriched diet delayed PCa progression at early stages by decreasing high-grade lesions, proliferation rate, and frequency of epithelial AR-positive cells in TRAMP mice [92].

In addition to AR signaling in PCa, the occurrence of alternative splicing of its transcripts is one of the main mechanisms implicated in the progression towards castration-resistant PCa as well as in resistance to AR-targeted therapies [93]. Among the AR variants, the AR-7 splicing variant (AR-V7) is one of the best characterized and is not targeted by most of the available AR signaling inhibitors (ARSIs). AR-V7 is common in metastatic PCa (75%) and is rare in early-stage disease (<1%), suggesting that its expression adaptively increases in tumors exposed to ARSIs [94]. A study that performed AR-V7 knockdown using siRNA in 22Rv1 cells, which abundantly express this variant, re-sensitized them to Enzalutamide [95]. Despite not being the only factor in determining castration-resistance, this AR variant may predict resistance to therapy and treatment failure [96]. Consequently, compounds targeting AR variants have emerged as a focus of research. We recently reported that DHA decreases AR-FL and AR-V7 expression in 22Rv1 cells as well as AR target genes [49]. However, we did not find any in vivo study reporting the effects of DHA on AR-V7, which is a gap in the literature to be filled.

Taken together, the literature supports androgen pathway suppression by DHA under different conditions, which are shared between in vitro and animal data to some extent, even though most in vivo studies used DHA-enriched supplementation, which compromises the interpretation of DHA’s exclusive effect. Also, it highlights the need for studies adopting new strategies to combine DHA with chemotherapy already used in patients under androgen deprivation, such as Flutamide, Bicalutamide, Nilutamide, Abiraterone, and Enzalutamide. The call for new studies using a DHA-only diet to better determine whether its anti-PCa effect does not rely on the combination with other omega-3 in vivo and patients is also in prospect. Importantly, the main challenge when considering combining the current therapies in PCa treatment is to ensure the controlled intake of omega-3 regarding frequency and concentration required to exert any effect on the tumor growth. In healthy humans, DHA seems to reach a concentration plateau in the blood after one month of supplementation that can be comparable with in vitro studies [97]. Considering this, one possible approach for patients responsive to androgenic ablation is an interventional trial to combine daily DHA intake in soft gels alongside ARSIs treatment and assessing tumor growth using imaging tools such as PET-SCAN or PET-PSMA, patient disease-free rate, and sustained PSA decrease. Additionally, there is a lack of studies showing whether patients expressing AR-FL or AR-V7 that were previously treated with ARSIs can be responsive to DHA intake with an improved outcome. Although studies using AR genetic editing (such as knockdown assays) are still required to confirm the DHA effect on androgenic signaling, it was reported that DHA also decreased cell growth of AR-negative cells. Therefore, it is reasonable to suggest that this fatty acid has plenty of mechanisms which do not depend on the androgenic background exclusively. Additionally, despite the evidence of DHA’s protective role in non-tumor cells, there is a lack of studies on humans, making it very difficult to determine the length of administration, proper concentration, and the timeframe.

### 5.2. Nuclear Receptors

In addition to AR, another well-known association between DHA and the regulation of nuclear receptors signaling is with the PPARs. In PCa, the role of PPARs is still under debate, mainly that of PPARγ because it may either favor PCa or act as a tumor suppressor. In the early stages, PPARγ overexpression is associated with the gland protection against cancer [98]. In pathological stages, it exhibits gene amplification in metastatic PCa compared to primary sites [99] and contributes to de novo lipogenesis [100], which is one of the key pathways in PCa carcinogenesis [58,60]. By inhibiting PPARγ with warfarin in mice, AR signaling was also suppressed [101]. However, this effect seems dependent on a PPARγ splice variant, specifically PPARγ1, while PPARγ2 exerts the opposite effect [102]. Accordingly, a rise in PPARγ1 expression in a PTEN-null PCa murine model enhanced tumor growth [103]. In this matter, stratification of PCa patients according to PTEN status could improve the outcome in future investigations that combine PPARγ1 inhibitors (such as thiazolidinediones, already under clinical trial) and DHA intakeInterestingly, DHA administration could be also considered in combination with PPARs agonists troglitazone, ciglitazone, and rosiglitazone which were reported as protective against PCa [104,105,106], while antagonists, specifically of PPARγ, have also been screened for a new therapeutic strategy [107].

DHA is a natural ligand of PPARγ, PPARδ, and PPARα, whereby it triggers oligodendrocyte differentiation [108] and pro-apoptotic signaling [109,110,111], decreases cell invasion [112], and causes the attenuation of inflammation [113,114] in different cell types. O’Flaherty et al. [115] showed that DHA itself or its 15-LOX-metabolites elicited a pro-apoptotic effect on PC3, LNCaP, and DU145 cells through the PPARγ/syndecan-1 axis. A limitation of this study is that the authors validated this only in the PC3 cell line. We have reported that regardless of their androgen status (AR-positive or AR-negative), tumor cells and non-tumor bearing malignant alterations showed nuclear receptors modulation and their co-regulators expression by DHA at 100 µM after 48 h [9]. These findings also suggested that the DHA’s ability to modulate such nuclear receptors expression decreases alongside PCa progression because in non-tumor PNT1A a higher number of regulated genes was found compared to castration-resistant 22Rv1 and PC3, the latter being the least responsive. In AR and PTEN positive cells, such omega-3 decreased *PPARG1A* and *PPARG1B* (PNT1A and 22Rv1, respectively) gene expression, whereas it upregulated *PPARG1A* in AR- and PTEN-negative cells [9]. In addition, it increased *PPARG* in PNT1A but not in tumor cells [9]. Lack of *PPARG1A* and *PPARG1B* was related to tumor suppression due to lipid and mitochondrial metabolism impairment, and *PPARG* upregulation in benign conditions was linked to its protective function [98]. Importantly, one of the limitations of this study is the lack of validation at protein level or in translocation assays. Like PPARs’ role in PCa, the direct effect of DHA on PPARs, including PPARγ, is not well defined and may differ according to the androgenic background. However, it was reported that ciglitazone decreases AR due to PPARγ in the androgen-independent cell C4-2 [105]. This highlights challenges but also suggests new possibilities for treatment, since DHA affects both PPARs and AR signaling, which has not been investigated yet.

Our group also demonstrated that a DHA-enriched diet reduces the frequency of epithelial and stromal glucocorticoid receptor (GR) positive cells in the prostate of TRAMP mice, which were linked to the decrease in cell proliferation [92]. Overexpression of GR is a common outcome of androgen deprivation therapy and results in the upregulation of several androgen-regulated genes and disease progression, indicating that GR may be a promising target in PCa therapy [116,117]. The use of glucocorticoids is concomitant with medications in the course of PCa treatment and may affect the tumor microenvironment (TME), specifically fibroblasts, promoting resistance to therapy [118]. Therefore, this reiterates that the DHA combination with AR-suppressing therapy could improve the outcome, but this remains to be investigated.

DHA is also known to regulate the activity of other nuclear receptors such as retinoid X receptor (RXR) and liver X receptor (LXR) [119,120,121], but the modulation of signaling pathways of these receptors by DHA in normal and cancer condition is largely unknown in the prostate, representing an opportunity for further exploration and therapeutic strategies.

### 5.3. Metabolism

AR and nuclear receptors signaling have a direct impact on cell metabolism, mainly the lipid metabolism, which undergoes reprogramming alongside PCa progression [60]. The fatty acid synthesis, also known as de novo lipogenesis, is the process whereby cells synthesize palmitate from acetyl-CoA exported from mitochondria and is an early hallmark of prostate carcinogenesis [60], often due to fatty acid synthase (FASN) overexpression [122,123]. The increased fatty acid synthesis is required by several cell functions, mainly membrane production [124], cell cycle progression [124], and intracellular signaling [125]. In PCa, lipogenic enzyme genes (such as FASN; Acetyl-CoA Carboxylase, ACC; and ATP citrate lyase, ACLY) show their expression regulated by AR via sterol regulatory element-binding protein (SREBP) [126,127]. Moreover, FASN was reported to co-localize with AR-V7 in castration-resistant PCa, and lipogenesis inhibition decreased cell and tumor growth [58]. In addition to their increased synthesis, fatty acids are also rapidly oxidized compared to glucose in PCa. Palmitate uptake is approximately 20 times higher than glucose, β-oxidation being the main source of energy in PCa [128,129]. This is supported by evidence that used Etomoxir, an inhibitor of long-chain fatty acid transport into mitochondria via carnitine palmitoyltransferase 1 (CPT-1), which led to cell death and decreased xenograft tumor growth in nude mice [128]. Interestingly, lipid uptake is controlled by androgens, which is reflected in the different landscapes of fatty acid membrane transporters among the androgenic backgrounds found in PCa [130]. Androgens enhance lipid uptake in AR-positive cells, especially fatty acids, cholesterol, and low-density lipoprotein particles [130].

Accordingly, in the past decades, several studies have revealed the role of a high-fat diet in PCa initiation and progression, which points to diabetes and obesity as potential risk factors [61,131,132,133,134,135,136]. In this context, not only the amount but also the type of lipids ingested may exert different effects on the prostate [137]. It was shown that saturated fatty acids drive PCa via the MYC program [61], while PUFAs seemed to exert the opposite effect, as already discussed. Given that PCa cells can take up DHA, it is reasonable to assume lipid deregulation to some extent. DHA incubation decreased the palmitic acid uptake in PCa, which is essential to support the lipid metabolism in tumors [138] and may be one of the multiple mechanisms whereby such omega-3 decreases cell proliferation. It also downregulated lipogenic genes such as *FASN*, *P-SREB*P, and *M-SREBP* in breast [139] and PCa cells [49]. Findings from our research group revealed that DHA at 100 µM after 48 h decreased the proliferation of PNT1A, 22Rv1, and PC3 cells associated with lipid accumulation, ROS overexpression, mitochondrial damage, and cell cycle arrest [9,140]. These effects were followed by regulation of several genes related to metabolism, response to lipids, stress, and hormones, but in distinct expression patterns according to androgenic background [9,140]. A study with a PC3 sub-population expressing markers of epithelial–mesenchymal transition (EMT) showed that cells with high invasiveness and low metastatic potential have DHA accumulation associated with decreased proliferation rate, whereas others with increased proliferation, low invasiveness, and high metastatic potential did not [141]. This difference was related to the increase of CPT-1 expression as well as β-oxidation, probably a mechanism to supply cell demands and eliminate pro-oxidant molecules, like DHA. The increase of lipid droplets is a known antioxidant mechanism [142], mainly against PUFAs increase which may trigger ferroptosis [62]. Accordingly, we have recently reported that DHA was incorporated as triacylglycerols and cholesterol esters in lipid droplets in LNCaP, C4-2, and 22Rv1 cells [49]. This accumulation was followed by deregulation of several genes related to lipid metabolism, a decrease in FASN expression, and metabolism reprogramming due to the decrease of lipogenesis from glucose [49]. However, these cells increased complete oxidation of glucose into CO_2_ and palmitate oxidation, except for 22Rv1, which preferably oxidized DHA [49]. One of the main findings of our study was that the impairment in lipid synthesis did not rely on AR expression, since its overexpression did not rescue lipogenesis from glucose [49]. This is relevant because it could support a new investigation in the CRPC stage, especially the combination of Enzalutamide with DHA. Conversely, in PC3 cells DHA increased de novo lipogenesis from glucose and glutamine in DHA-enriched media [25,140]. This suggests that the metabolic adjustment due to DHA uptake may depend on the androgenic background. Currently, TVB-2640 (Denifanstat), a FASN irreversible inhibitor, is under clinical trial for PCa (NCT03808558) and statins (NCT00572468 and NCT04026230), HMG-CoA reductase inhibitors belonging to the cholesterol biosynthesis pathway. Taken together, our data strongly support that DHA could synergize with them and improve the outcome. Other pharmaceutical interventions to target lipid metabolism are under assessment as pre-clinical trials, like IPI-9119 (FASN inhibitor), PF-05175157 (ACC1/ACC2 dual inhibitor), A939572 and CAY10566 (SCD1 inhibitors), Fatostatin and Betulin (SREBP-inhibitor signaling), and NB-598 (SQLE inhibitor) that could benefit from DHA intake and its synergistic potential. An important addition to the field would be to investigate the impact of DHA supplementation on the regulation of elongases and desaturases that can favor PCa. Taken together, this collective evidence indicates that molecular adjustments in lipid metabolism occur and that fatty acids are differentially used in each stage of PCa progression. Furthermore, these findings also showed that lipid metabolism may be the Achilles heel in PCa, and they have been explored as a therapeutic approach [143].

Mitochondria are also altered in PCa. They show increased pleomorphism [144], yield [145,146], bioenergetics and biogenesis gene overexpression [147], tricarboxylic acid adjustments [145], and remodeling of oxidative phosphorylation (OXPHOS) [148], being also related to progression to androgen independence [149]. Therefore, mitochondria have been investigated as a potential target not only in prostate but also in many other cancers [150,151]. In an in vitro study, DHA exerted a preventive property in PNT1A epithelial prostate cells via bioenergetic modulation by impairing the bioenergetic reserve capacity, increasing mitochondria area, and enhancing the antiproliferative effect of melatonin [24]. In PNT1A and CRPC cells, DHA rewired the cell metabolism by impairing mitochondrial function and its spare capacity, decreasing ATP production, inducing membrane hyperpolarization, and increasing superoxide anion production [25,140]. In a DHA-enriched medium, phosphatidylglycerol, a precursor of cardiolipin, increased in CRPC cells as well as its unsaturation status in mitochondrial membrane, changing its composition and inducing organelle fragmentation, which supports the rise in oxidative damage [25,140]. The ability to reduce the spare capacity (also known as bioenergetic reserve capacity) is of particular interest in cancer because it might be a strategy to sensitize cells to additional compounds. Indeed, DHA was reported to improve the antiproliferative effects of chemotherapeutic compounds in several cancers [152,153,154]. Therefore, this body of evidence suggests that DHA affects critical cell metabolism pathways in a molecular context manner, as illustrated in Table 1. Moreover, it sheds light on new therapeutic strategies targeting lipid metabolism deregulation by DHA in PCa, mainly in the castration-resistant form.

### 5.4. Modulation of Cell Death

The aforementioned mechanisms whereby DHA may exert its antitumor effect against PCa culminate in cell growth suppression, which can be due to cell death. In PCa, PUFAs’ uptake and their accumulation mainly in membranes were reported to increase oxidative damage, inhibit glutathione peroxidase 4 (GPX4), and trigger ferroptosis [62]. Along this line, DHA was able to enhance the effect of ferroptosis inducers in vivo and in vitro by increasing ROS and lipid peroxidation, regardless of its receptor GPR120 [155]. Accordingly, resistant cells to erastin, a ferroptosis inducer, were sensitized by DHA [156]. Despite being insightful, this study lacked a proper panel of ferroptosis protein markers and only observed DHA effects at high concentrations and mostly combined with ferroptosis inducers. It did not evaluate whether AR plays a role even though it used PCa cell lines with a distinct androgenic background. Additionally, this study used a xenograft model by inoculating PC3 cells in mice, which excludes the DHA role in the tumor microenvironment. It would be important to assess whether the formation of anti-inflammatory intermediates derived from DHA could modulate immune cells and tumor burden. Interestingly, in AR-negative cells, which are resistant to several treatments, DHA was reported to induce cell death. In PC3 and DU145, both metastatic cells expressing mutant p53, DHA inhibited AKT/mTOR mediated by ROS, which seems to lead to cell death by apoptosis, given the modulation on PARP expression [23]. However, crucial markers of cell death such as caspase cascade were not assessed to strengthen the mechanism. Sun et al. [157] reported in DU145 that at 50 µmol/L for 24 h DHA modulated several apoptosis-related genes involved in the p53 pathway, as well as TNF signaling, AKT, and the mitochondrial-related pathway, suggesting the modulation of both extrinsic and intrinsic apoptosis. However, this was not confirmed at protein level and cascade activation. DHA-induced apoptosis in both PC3 and DU145 was also related to YAP phosphorylation and cytoplasm translocation via the FFAR1/FFAR4-Gαs-PKA-Hippo pathways [37]. In the same study, this was not observed for androgen-sensitive LNCaP cells, which strengthen the different mechanisms triggered by this omega-3 in different molecular contexts. However, others reported that DHA selectively inhibited AKT^T308^ but not AKT^S473^ phosphorylation, altered PIP3 and AKT protein localization, and induced apoptosis through AKT downstream BAD (BCL2 Associated Agonist Of Cell Death) inhibition regardless of the androgenic background [57]. These findings suggest that DHA can induce cell death regardless of AR signaling suppression. In TRAMP mice, a DHA-enriched diet (4 g ± 0.3 g of omega-3/day) during early (8 to 12 weeks of age) and late stages (8 to 18 weeks of age) of PCa progression reduced cell proliferation and increased apoptosis in the former, while in the latter it downregulated pyroptosis and upregulated necroptosis [158]. The entire mechanism whereby DHA triggers cell death is not clear, and proper rescue experiments are lacking in the current studies as well as additional animal studies. Nevertheless, one possible and interesting contribution to the literature would be the combination of PARP inhibitors already used in chemotherapy for PCa, like Olaparib (PROfound Trial, NCT02987543) with DHA to enhance tumor growth suppression. This would be valuable for CRPC, which lacks effective therapy. A simple in vitro test would be to combine DHA with PARP inhibitors simultaneously to assess the synergistic effect. Pre-incubation with DHA should be also considered to understand whether it can sensitize cells to the inhibitors. This would support further in vivo approaches, which are relevant to support clinical trials, since Olaparib is already used in patients. Moreover, correlation analysis of survival and disease-free rates in patients with PCa under Olaparib treatment with high and low intake of DHA through diet or nutritional supplementation would be insightful. [177Lu]Lu-PSMA-617 (Pluvicto) is a radioligand therapy approved by FDA (VISION Trial—NCT03511664) used against PCa that induces apoptosis and necrosis via DNA damage that could be combined with DHA. Another chemical that is under clinical trial is Navitoclax (NCT02520778), a BCL-2 family inhibitor that showed modest efficacy in Phase II, which could have the outcome improved by DHA.

### 5.5. Modulation of Prostate Tumor Microenvironment

PCa cells are mostly derived from epithelium, but the surrounding stromal cells firmly support the tumor growth through several mechanisms, including metabolic exchange and immune evasion. The tumor microenvironment (TME) is a term that refers to the highly complex structure that comprises the tumor and the surrounding non-tumor cells, such as stromal elements like fibroblasts and a diversity of immune cells that collectively impact tumor onset and progression [159,160]. Macrophages are monocytes that can be undifferentiated (M0) or polarized into M1, pro-inflammatory, or M2-like, which have anti-inflammatory properties. DHA undergoes enzymatic reactions to generate derived metabolites that can be classified into maresins, resolvins, and protectins, which can modulate immune cells [161,162]. Additionally, DHA content increases with diet in macrophages, lymphocytes, and neutrophils [161]. Cultured resident peritoneal macrophages can uptake DHA and incorporate it into phospholipids such as phosphatidylcholine and phosphatidylethanolamine in the resting state, which change upon activation [163]. Likewise, DHA was able to enhance natural killer cells’ antitumor functions and their interferon gamma production, decreasing tumor growth in a melanoma model [164].

A high-fat diet has been investigated as a relevant factor that engages PCa initiation or favors its progression due to the generation of a favorable microenvironment [61,132,133,134,135,136]. However, a body of evidence shows that omega-3 fatty acids may exert the opposite effect [165,166,167], suppressing tumor growth instead. DHA is known for its anti-inflammatory function, and its administration can modulate the microenvironment by decreasing inflammation, which is frequently linked to PCa development [168,169]. Its anti-inflammatory effect is mainly produced along with its oxygenation, and, in PCa, resolvins from class D (RvD1, RvD2, RvD3, RvD4, and RvD5) have caught the attention. RvD1 and RvD2 inhibited tumor-associated macrophages (TAMs) and macrophage polarization into M1, whereas they stimulated M2a, a phenotype that attenuates inflammation [170]. In vivo studies that exclusively use DHA in their experimental design are scarce. Instead, most of the literature provided a blend of omega-3 and reported immune cell modulation in PCa models [166,167,171,172]. In MycCaP allographs, the ω-3 diet reduced M2 TAM infiltration and led to a reduction of tumor growth through inhibition of angiogenesis and reversion of M2-suppression of T-cell activation [167,171]. Interestingly, these effects seem to be mediated by GPR120, since MycCaP allographs in GPR120^−/−^ mice are insensitive to the antitumor effects of a ω-3 diet, and knockout of GPR120 in M2-polarized macrophages reverses the inhibitory effects of DHA on M2-related gene expression and cancer cell migration [165,171]. Accordingly, we have reported that the DHA-enriched diet administered to TRAMP mice reduced CD4^+^ T-cells and M2-like macrophages while increasing CD8^+^ T-cell infiltration at a late stage of progression, which suggests the PUFA protective role [158].

Stromal cells play a pivotal role in PCa progression [173]. Among them are fibroblasts [159] since they can support the metabolic demands of PCa cells [174]. In PCa, DHA was shown to inhibit the differentiation of fibroblasts into myofibroblasts, the active form of the former and usually referred to as cancer-associated fibroblasts (CAFs), which decreased PC3 cells’ invasiveness [175]. When exposed to DHA, PC3 cells display impairment of LPS-induced metastatic activities, mainly due to downregulation of interleukin-6 (IL-6) and interleukin-8 (IL-8) production, reduced PTGS2 expression, and inactivation of AKT signaling [176]. The effects of DHA on TME were summarized in Figure 2.

### 5.6. Combination of DHA with Other Therapies

The mechanisms underlying DHA antitumor effects have been investigated in combination with other compounds to potentiate the inhibition of tumor and cell growth. The omega-3 was shown to enhance the effects of ARSIs, including in darolutamide-resistant 22Rv1 [177]. As we have mentioned, DHA was reported to amplify the melatonin antiproliferative effect in PNT1A cells [24]. It was shown to synergistically enhance the cytotoxic effect of docetaxel in PCa cells through increased apoptosis by suppression of genes involved in the NFkβ pathway [178]. Also, DHA combination with ciprofloxacin conjugates increased the cytotoxicity in DU145 cells compared to LNCaP [179] and enhanced the antitumor activity of polymeric conjugates grafted with cabazitaxel in AR-negative cells [180]. DHA intake alone was shown to decrease tumor growth by 80% in the 22Rv1 xenograft model as well as the viability and diameter of MSK-PCa3 organoids [49]. Also, the omega-3 administration was shown to sensitize several tumors, like breast, lung, prostate, and lymphoma, to different chemotherapeutic agents [24,181]. Clinical studies that assessed chemotherapy combined with fish oil administration (2.2 g EPA + 240 mg DHA or 2.2 g EPA + 500 mg DHA/day during 4 cycles) revealed that it may improve the outcome in advanced non-small cell lung cancer, including survival rate [182]. Regarding DHA administration only, this omega-3 at 1.8 g/day previous to chemotherapy (1–7 days) and along with it (5 months), increased its plasma levels associated with improved outcome and overall survival in metastatic breast cancer patients [183]. It is also important to mention that these studies confirmed the non-toxic effects of DHA or fish oil administration [182,183]. As the chemotherapeutic agent, DHA is systematically distributed when administered, but its selective action in tumors rather than normal cells was reported and revised [181]. Despite evidence that has suggested its safety, there is a lack of evidence concerning PCa that requires further investigation.

**Table 1:** DHA effects on gene expression of prostate cancer cells. The symbols indicate increased (↑) or decreased (↓) gene expression. The main functions of proteins coded by the genes: 1—Cell Proliferation; 2—Cell Death; 3—Inflammation; 4—Metabolism; 5—Hormone Signaling; 6—Metastasis; 7—Other. Legend: *AR*-FL—Full-Length androgen receptor; *ATF3*—Activating Transcription Factor 3; *BAX*—BCL2-associated X Protein; *CASP1*—Caspase 1; *CASP 3*—Caspase 3; *CASP9*—Caspase 9; *CCNA2*—Cyclin A2.; *CCND2*—Cyclin D2; *CIDEA*—Cell Death-inducing DFFA-like Effector A; *DRG-1*—Developmentally Regulated GTP Binding Protein 1; *ERRFI1*—ERBB Receptor Feedback Inhibitor 1; *FADD*—Fas Associated Via Death Domain; *FASN*—Fatty Acid Synthase; *FKBP51*—FK506-binding Protein 51; *FOS*—Transcription Factor AP-1 Subunit C-Fos; *HDAC5*—Histone Deacetylase 5; *KLK3*—PSA; ODC—Ornithine Decarboxylase; *LTA*—Lymphotoxin Alpha; *MAX*—Transcription factor MAX; *MAP2K4*—Mitogen-Activated Protein Kinase Kinase 4; *NR0B1*—Nuclear Receptor Subfamily 0 Group B Member 1; *PPARG*—Peroxisome Proliferator Activated Receptor Gamma; *PPARGC1A*—PPARG Coactivator 1 Alpha; *PPARGC1B*—PPARG Coactivator 1 Beta; *PTGS2*—Prostaglandin-Endoperoxide Synthase (COX2); *RIPK1*—Receptor Interacting Serine/Threonine Kinase 1; RORA—RAR Related Orphan Receptor A; *TRAF3*—TNF Receptor Associated Factor 3; TMPRSS2—Transmembrane Serine Protease 2; *TP53*—Tumor Protein 53; *TNFRSF11A* (RANK)—TNF Receptor Superfamily Member 11A; *WAF/CIP1*—p2—Cyclin Dependent Kinase Inhibitor 1A; IL6 – Interleukin 6; IL10—Interleukin 10; AKT1—AKT1 Kinase; NKX3.1—NK3 Homeobox 1.

GENE	DHA EFFECT	MODEL (S)	FUNCTION	REFERENCE
*AKT1*	**↓**	LNCaP, DU145	1, 2, 4	[178]
*AR*	**↓**	DU145	1–6	[86]
*ATF3*	**↑**	PC3	2	[22]
*BAX*	**↑**	DU145	2	[157]
*CASP1*	**↑**	DU145	2	[157]
*CASP3*	**↑**	DU145	2	[157]
*CASP9*	**↑**	DU145	2	[157]
*CCNA2*	**↓**	PC3	1	[22]
*CCND2*	**↑**	PC3	1	[22]
*CIDEA*	**↑**	DU145	2	[157]
*DRG-1*	**↓**	LNCaP	6	[86]
*ERRFI1*	**↑**	PNT1A	1, 4, 5	[9]
*FADD*	**↓**	LNCaP, DU145	2	[178]
*FASN*	**↓**	PC3	3	[22]
*FKBP51*	**↓**	LNCaP cells	1, 3, 5	[86]
*FOS*	**↓**	PNT1A, 22Rv1	1	[9]
*HDAC5*	**↑**	22Rv1	1, 3	[9]
*IL6*	**↑**	PC3	3	[22]
*IL10*	**↑**	PC3	3	[22]
*KLK3*	**↓**	LNCaP	7	[86]
*LTA*	**↑**	DU145	2	[157]
*ODC*	**↓**	LNCaP cells	2, 5	[86]
*PPARG*	**↑**	PNT1A	1, 4	[9]
*PPARGC1A*	**↓**	PNT1A, PC3	1, 4	[9]
*PPARGC1B*	**↑**	22Rv1	1, 4	[9]
*MAX*	**↓**	LNCaP, DU145	1, 2	[178]
*MAP2K4*	**↓**	LNCaP, DU145	1, 2, 6	[178]
*NKX3.1*	**↓**	LNCaP	1, 6	[86]
*NR0B1*	**↑↓**	PNT1A, 22Rv1, PC3	4, 5, 7	[9]
*PTGS2*	**↑**	PC3	3	[22]
*RIPK1*	**↓**	LNCaP, DU145	2	[178]
*RORA*	**↑**	22Rv1	4, 5	[9]
*TNFRSF11A*	**↓↑**	LNCaP, DU145	2, 3, 6	[178]
*TMPRSS2*	**↓**	LNCaP	1	[86]
*TP53*	**↑**	DU145	2, 6	[157]
*TRAF3*	**↓**	LNCaP, DU145	2, 3	[178]
*WAF/CIP1*	**↑**	PC3	1	[22]

## 6. Conclusions, Limitations, and Future Directions

The main gaps found in the literature to be filled, and potential translational approaches, are summarized in Table 2. Taken together, we concluded that DHA may trigger a plethora of mechanisms in PCa which cannot be considered alone but seem to work in an orchestrated manner towards the decrease of cell or tumor growth. In particular, the underlying mechanisms include not only androgenic signaling, which is often a target in the current therapies, being a promising feature since such a strategy may lead to recurrence in a more aggressive phenotype. Moreover, it is a PUFA commonly found in the diet, with an accessible cost through diet or nutritional supplementation and with insignificant side effects compared with conventional therapy, being often recommended due to its health benefits in cancer-free patients. In this context, even if not fully efficient by itself in suppressing tumor growth, DHA was shown to improve chemotherapy, which was shown for the mixture of omega-3 in rodents. Although it has promising effects, one limitation for drawing solid conclusions on PCa is the scarce evidence in vivo of DHA-only experiments, which must be addressed in future investigations to assign its specific effects. Also, more animal studies are required to better elucidate the role of DHA in different stages of disease, including undifferentiated, neuroendocrine-like and castration-resistant tumors with distinct mechanisms of resistance to androgen deprivation therapy.

## Figures and Tables

**Table 2:** Gaps in literature and potential translational applications. Legend: AR—androgen receptor; ARSIs—androgen signaling inhibitors; DHA—docosahexaenoic acid; PCa—prostate cancer; PPARs—peroxisome proliferator-activated receptors; GR—glucocorticoid receptor; TME—tumor microenvironment; HOT—inflamed tumor microenvironment; COLD—immune cell depleted tumor microenvironment.

MECHANISM	MAIN GAPS FOUND IN THE LITERATURE	POTENTIAL TRANSLATIONAL APPROACH
***Androgen signaling***	In vivo studies; in vitro combination with ARSIs simultaneously or considering DHA pre-incubation; lack of study using AR genetic editing for validation	Combination of DHA intake with ARSIs currently available
***Nuclear Receptors***	Determination of whether PPARs are protective or promote PCa; in vivo and in vitro studies with DHA combined with PPARs inhibitors; association with androgenic background	Combination of DHA intake with PPAR inhibitors in patients stratified according to PTEN expression; combination of DHA with drugs that regulate GR.
***Metabolism***	Combination with lipogenesis and cholesterol biosynthesis inhibitors in vivo and in vitro; lack of carbon-labeled experiments to evaluate the metabolic flux	Synergistic effect of DHA intake with lipid metabolism inhibitors under clinical trial
***Cell Death***	In vivo assays; lacks assessment of combination either in vitro or in vivo with cell death inhibitors with proper controls	Synergistic effect of DHA intake with cell death inducers under clinical trial, simultaneously or as pretreatment
***TME***	Co-culture assays to validate hypothesis; functional assays ex vivo to validate lymphoid and myeloid function; specific assessment of DHA in COLD and HOT TME	Stratification of patients according to inflammatory infiltrates; combination of DHA intake with the current therapy

## Data Availability

No new data were created or analyzed in this study.
